# Investigation of the distribution of inguinal lymph nodes and delineation of the inguinal clinical target volume using ^18^F-FDG PET/CT

**DOI:** 10.1186/s12885-024-13015-w

**Published:** 2024-10-10

**Authors:** Jia-Li Han, Yan-Ge Qi, Jia-Ling Liu, Xia Yan, Wan-Chun Zhang, Ling Yuan, Xin-Zhong Hao, Jian-Bo Song, Si-Jin Li

**Affiliations:** 1 Department of Nuclear Medicine, First Hospital of Shanxi Medical University, Taiyuan, Shanxi 030001 China; 2 Shanxi Bethune Hospital, Third Hospital of Shanxi Medical University, Shanxi Academy of Medical Sciences, Tongji Shanxi Hospital, Taiyuan, Shanxi 030032 China; 3 Shanxi Provincial Key Laboratory for Translational Nuclear Medicine and Precision Protection, Taiyuan, Shanxi 030006 China; 4 Department of PET/CT, Cancer Hospital Affiliated to Shanxi Medical University, Taiyuan, Shanxi 030013 China

**Keywords:** Pelvic and perineal cancer, Inguinal lymph nodes metastases, Clinical target volume, ^18^F-FDG PET/CT, Intensity-modulated radiotherapy

## Abstract

**Objective:**

Radiotherapy is a crucial treatment modality for pelvic cancers, but uncertainties persist in defining the clinical target volume (CTV) for the inguinal lymphatic drainage region. Suboptimal CTV delineation may compromise treatment efficacy and result in subpar disease control. This study aimed to investigate and map the distribution of lymph node metastases (LNM) in the groin area to facilitate an improved and detailed CTV definition using ^18^F-fluorodeoxyglucose positron emission tomography/computed tomography (^18^F-FDG PET/CT).

**Methods:**

Inguinal LNM in patients with biopsy-proven pelvic malignancies were identified using ^18^F-FDG PET/CT scan. The longitudinally nearest axial plane was determined based on six typical bony landmarks, and the axial direction relative to the femoral artery of LNM was recorded. The distances from the LNM to the nearest edge of the femoral artery were measured on the axial plane. An optimal margin to cover 95% of LNM was estimated to develop contouring recommendations.

**Results:**

In this study, 500 positive LNM were identified by ^18^F-FDG PET/CT among 185 patients with primary pelvic malignancies. Relative to the femoral artery, lymph nodes were distributed laterally (10:00–11:00, *n* = 35), anteriorly (12:00–1:00, *n* = 213), and medially (2:00–4: 00, *n* = 252). For CTV delineation, the recommended distances from the femoral artery on the SFH were lateral 19 mm, anterior 19 mm, and medial 25 mm; on the SGT were lateral 26 mm, anterior 20 mm, and medial 25 mm; on the SPS were lateral 28 mm, anterior 29 mm, and medial 26 mm; on the IPS were anterior 29 mm and medial 28 mm; on the IIT were anterior 27 mm and medial 27 mm; on the ILT were anterior 25 mm and medial 23 mm. Use interpolation to contour the area between six axial slices, including any radiographically suspicious LNM.

**Conclusions:**

Using ^18^F-FDG PET/CT, we investigated the distribution pattern of inguinal LNM and propose a more comprehensive guideline for inguinal CTV delineation.

**Supplementary Information:**

The online version contains supplementary material available at 10.1186/s12885-024-13015-w.

## Introduction

Several pelvic malignancies, including carcinomas of the vulva, distal rectum, anal canal, penis, vagina, and cervix involving the distal one-third of the vagina, have the propensity to metastasize to the inguinal lymph node [1]. Such metastases are often associated with locoregional recurrence and poor overall survival (OS). To address this issue, multiple consensus guidelines have recommended the use of definitive external beam radiotherapy with concurrent chemotherapy [2, 3]. In contrast to conventional radiotherapy, intensity-modulated radiation therapy (IMRT) delivers highly conformal radiation therapy, offering superior coverage of target structures while sparing adjacent organs, such as the bowel, bladder, bone, and muscle [4, 5]. However, the utilization of IMRT requires comprehensive anatomic knowledge of lymph node metastases (LNM) and precise delineation of at-risk LNM regions to ensure optimal target coverage and minimize doses to nearby critical organs. Although there is a consensus on the definition of the pelvic nodal clinical target volume (CTV) in existing guidelines and atlases [6], guidelines for contouring the optimal CTV of inguinal LNM remain inconclusive and lack precision [7–12] (summarized in Supplementary Table 1). Hence, to further enhance the efficacy of IMRT, a more precise delineation of the inguinal CTV is still necessary.

Currently, CT and MRI are the most commonly used modalities for the preliminary diagnosis and CTV delineation of inguinal LNM in clinical practice. Nevertheless, the sensitivity of CT and MRI in identifying LNM in terms of nodal size and morphology is limited, ranging from 24 to 78% [13, 14]. ^18^F-fluorodeoxyglucose positron emission tomography/computed tomography (^18^F-FDG PET/CT) obtains a unique combination of anatomic information from CT and metabolic functional information from PET. It has been found to maintain high specificity and significantly improve sensitivity (85-100%) in detecting LNM [15–17]. As a result, ^18^F-FDG PET/CT holds promising prospects for broader application in LNM detection and guiding radiation treatment planning [18–20].

The purpose of this study was to determine the distribution of the inguinal LNM through ^18^F-FDG PET/CT and map them according to the relationship of the femoral artery on corresponding axial slices of the CT images. Subsequently, we aimed to ascertain the optimal margin around the femoral artery to achieve complete LNM coverage and to propose evidence-based guidelines for inguinal nodal CTV delineation.

## Methods

Between 2010 and 2023, medical records and imaging information of patients with primary pelvic carcinomas who underwent ^18^F-FDG PET/CT at the First and Third hospitals of Shanxi Medical University were retrospectively reviewed by two experienced nuclear medicine experts and one radiation oncology expert. Patients with inguinal LNM were included, while those who had undergone therapy in the groin region or whose image quality was abnormal were excluded. The criteria for identifying metastatic nodes were as follows: nodes with a short-axis diameter greater than or equal to 10 mm in the short axis [21–23], nodes with a short-axis diameter less than 10 mm but exhibiting abnormal morphology (e.g., round in shape, irregular border due to extracapsular extension, loss of fatty hilum, or display of necrosis) on CT, or nodes showing ^18^F-FDG avidity greater than background liver on PET [24].

**Fig. 1:**
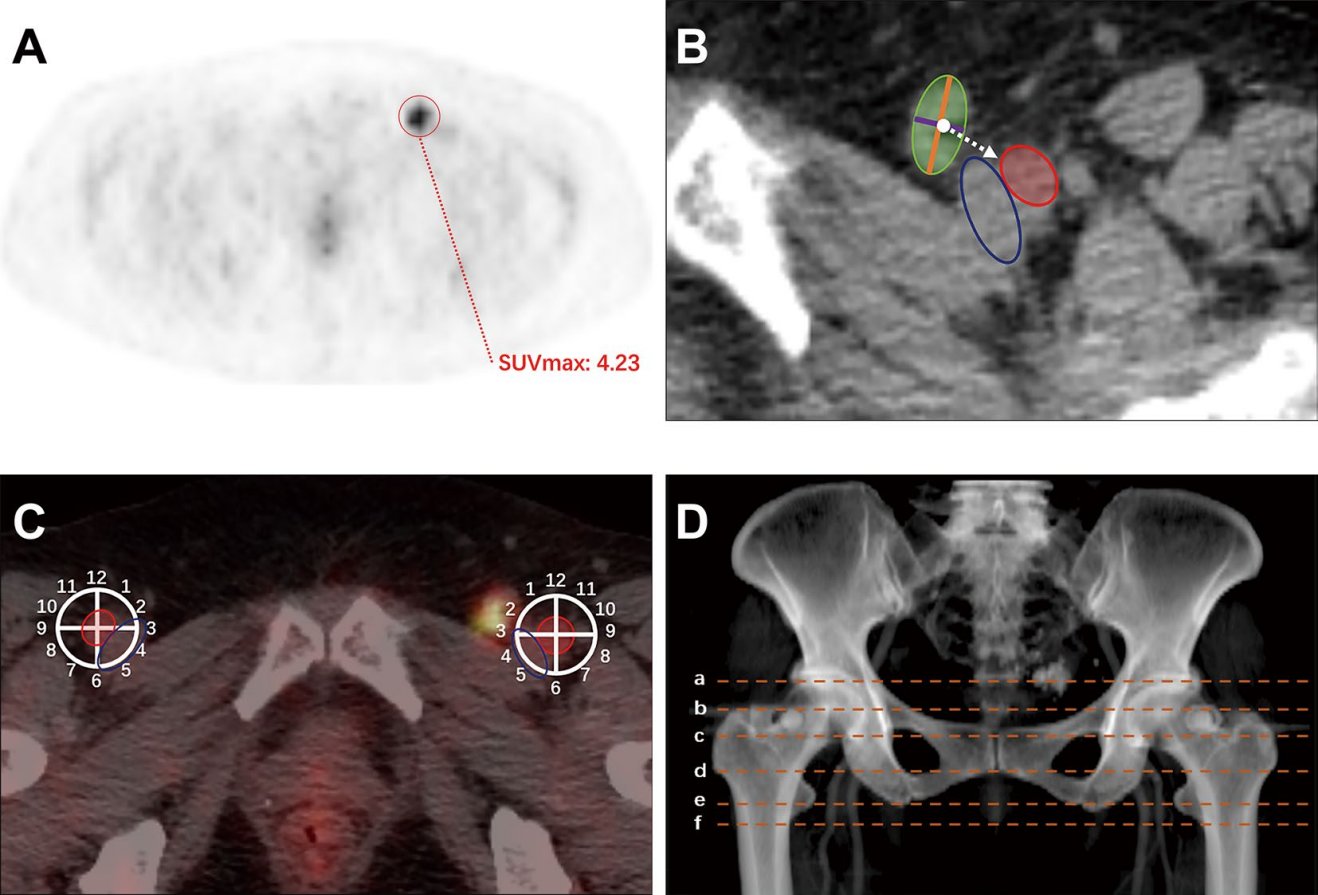
Method diagram. (**A**)The SUVmax of the metastatic lymph node was measured and recorded; (**B**) The short-axis diameter (purple line) and long-axis diameter (orange line) of the lymph node (green) as well as the nearest distance (white dotted arrow) from the center of the node to the femoral artery (red) were documented on the CT slice; (**C**) The orientation of the node was indicated by the clock face with 12:00 representing the anterior direction, 3:00 the medial direction, 6:00 the posterior direction, and 9:00 the lateral direction, relative to the center of the femoral artery (red) on the axial plane; (**D**) The node was recorded at the nearest level of 6 typical bony landmarks (orange dotted line) longitudinally: the level of the superior edge of the femoral head (**a**), the level of the superior edge of the greater trochanter (**b**), the level of the superior edge of the pubic symphysis (**c**), the level of the inferior edge of the pubic symphysis (**d**), the level of the inferior edge of the ischial tuberosity (**e**), and the inferior edge of the lesser trochanter (**f**)

The location, size, and maximum standardized uptake value (SUVmax) of inguinal LNM in the enrolled patients were documented. The SUVmax of the LNM was measured (Fig. 1A). The short and long axis diameters of the LNM, as well as the nearest distance from the center of the node to the nearest edge of the femoral artery, were determined on CT (Fig. 1B). The orientation of the LNM was indicated by the clock face, with 12:00 representing the anterior position, 3:00 medial, 6:00 posterior and 9:00 lateral, all relative to the femoral artery center on the axial plane (Fig. 1C). To increase the accuracy of LNM localization, the nodes were recorded on the nearest level of 6 typical bony landmarks longitudinally: the level of the superior edge of the femoral head (SFH), the level of the superior edge of the greater trochanter (SGT), the level of the superior edge of the pubic symphysis (SPS), the level of the inferior edge of the pubic symphysis (IPS), the level of the inferior edge of the ischial tuberosity (IIT), and the inferior edge of the lesser trochanter (ILT) (Fig. 1D). Additionally, the distances from the lymph node to the IPS and the ILT were measured on the 2D coronal plane.

Based on location information of LNM obtained from the PET/CT scans, two radiation oncology experts utilized the MIM system 7.1.2 to delineate all lymph nodes on the representative planning CT with a 5 mm diameter sphere contouring tool. Eventually, margins around the femoral artery were calculated on different axial planes and orientations, as well as from the inferior aspect, to cover 90% and 95% of the nodes, respectively.

## Results

### General information of patients and LNM

Our institutional database revealed that 228 patients with primary pelvic and perineal cancer had inguinal LNM detected by PET/CT. Patients with recurrent inguinal lymph nodes previously treated (*n* = 41), or incorrect body position at PET/CT imaging (*n* = 2) were excluded. Consequently, a total of 185 patients with 500 LNM were included in this study. Of these patients enrolled, 127 patients presented with unilateral LNM, and 58 patients exhibited bilateral LNM. The number of identified LNM was higher with ^18^F-FDG PET/CT compared to CT alone (500 vs. 306).

Out of the 500 lymph nodes, 468 were defined as metastatic based on increased FDG uptake. Among these, 274/468 (58.5%) lymph nodes with heightened FDG uptake were also defined as metastasis due to their morphology, whereas 32/306 (10.5%) lymph nodes with suspicious morphology did not exhibit enhanced FDG uptake. Moreover, 28/185 (15.1%) patients had a change in clinical stage due to the addition of PET. The mean SUVmax of LNM was 5.6 (range 1.0-38.4) and the mean SUVmean of the liver was 2.2 (range 1.1–4.3). The median short axis of LNM was 10 mm (range 4–48) and the median long axis was 14 mm (range 4–59). Detailed characteristics of patients and LNM are presented in Table 1.

**Table 1:** Patients and tumors’ characteristics

Characteristics	Value
**Age in years**,** median (range)**	57 (29–84)
**Sex**,** n (%)**	
Male	46 (25)
Female	139 (75)
**Body mass index (BMI)**,** median (range)**	23.6 (15.1–34.4)
Tumor types at clinical diagnosis n* (%)	
Cervical cancer	87 (47)
Rectal cancer	28 (15)
Penial cancer	25 (14)
Vulvar cancer	19 (10)
Vaginal cancer	14 (8)
Anal cancer	12 (6)
**Lymph node laterality**,** n (%)**	
Unilateral	127 (69)
Bilateral	58 (31)
**The imaging method of malignant node identify**, N* (%)	
Positively diagnosed by PET	468 (94)
Positively diagnosed by CT	306 (61)

*: *n* = 185 patients; *N* = 500 malignant lymph node

### Distribution of the LNM around the femoral artery in different axial planes

Relative to the femoral artery, 1% of LNM (*n* = 2) were located at 10:00, 6% (*n* = 33) at 11:00, 13% (*n* = 64) at 12:00, 30% (*n* = 149) at 1:00, 28% (*n* = 140) at 2:00, 15% (*n* = 75) at 3:00, and 7% (*n* = 37) at 4:00. The majority of LNM were situated medially (2:00–4:00, 50%) and anteriorly (12:00–1:00, 43%) to the femoral vessels, while very few were located laterally (10:00–11:00, 7%), and no lymph nodes were found posteriorly (5:00–9: 00).

The proportion of LNM in different axial planes varied: 11% were located at the level of SFH (*n* = 54), 18% at the level of SGT (*n* = 89), 36% at the level of SPS (*n* = 182), 22% at the level of IPS (*n* = 110), 7% at the level of IIT (*n* = 34), and 6% at the level of ILT (*n* = 31). The number of LNM at different locations is shown in Table 2.

**Table 2:** The number of LNM at different locations

Direction	Lateral		Anterior		Medial		
	10:00	11:00	12:00	1:00	2:00	3:00	4:00
SFH	0	5	3	4	2	13	27
SGT	1	21	18	16	2	23	8
SPS	1	4	20	63	70	22	2
IPS	0	1	8	41	44	16	0
IIT	0	1	6	14	12	1	0
ILT	0	1	9	11	10	0	0

**Acronyms**: SFH = the level of the superior edge of the femoral head; SGT = the level of the superior edge of the greater trochanter; SPS = the level of the superior edge of the pubic symphysis; IPS = the level of the inferior edge of the pubic symphysis; IIT = the level of the inferior edge of the ischial tuberosity; ILT = the level of the inferior edge of the lesser trochanter

**Fig. 2:**
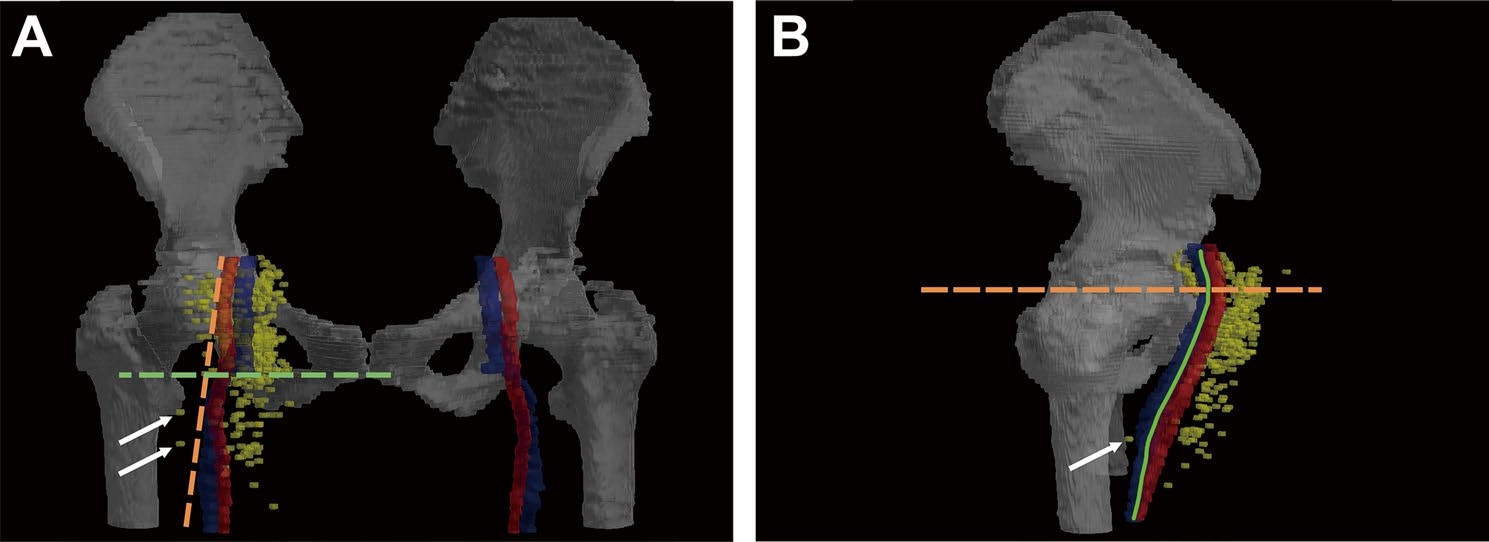
Overall distribution map of inguinal lymph nodes. Most of the lymph nodes are distributed in the medial (50%) and anterior (43%) regions relative to the femoral vessels. (**A**) The overall distribution of inguinal lymph nodes forms a funnel shape (pink dotted line); the lymph nodes (yellow dots) on the lateral side of the femoral vessels (outside the orange dotted line) are mostly located above the level of the inferior edge of the pubic symphysis (green dotted line), and only three nodes (indicated by white arrows) are distributed below the inferior edge of the pubic symphysis. (**B**) Except for some lymph nodes at 4:00 located above the level of the superior edge of the greater trochanter (orange dotted line) in the external iliac transition zone, there are no lymph nodes distributed behind the femoral artery (behind the green solid line)

The anterior and medial lymph nodes at the level of SPS accounted for the majority (*n* = 177, 35%), suggesting the distribution center of LNM. The overall distribution of LNM exhibited a decreasing trend from the center to the periphery. Only two lymph nodes were located at 10:00, and merely three lymph nodes were below the IPS level in the lateral direction (10:00–11:00). In the medial 4:00 direction, most lymph nodes (35/37, 95%) were situated at the level of SFH and SGT, which is where the external iliac vessels leave the bony pelvis and transition into the femoral vessels. No diseased node was detected below the IPS at 4:00 and just one node below the level of IIT at 3:00. The nodal distribution demonstrated a funnel-shaped pattern, which should be taken into account when outlining the CTV. The distribution of lymph nodes relative to femoral vessels and bone anatomical structures is illustrated in Fig. 2.

### Distance between the LNM and the different reference points

The median distance between LNM and the nearest edge of the femoral artery was 17 mm (range 3–43), and 30% of the lymph nodes were located more than 20 mm from the edge of the femoral artery. The distances from the center of the lymph nodes to the nearest edge of the femoral artery varied in four different directions. The median distances were 18 mm (range 6–30) laterally, 17 mm (range 3–43) anteriorly, and 18 mm (range 6–35) medially. The distances required to include 95% of the lymph nodes were 27 mm, 29 mm, and 26 mm, respectively, in the same lateral, anterior, and medial directions. Moreover, on the different axial planes, the distances between the lymph nodes and the femoral artery presented variations, displaying a certain regularity. As slices progressed from cranial to caudal level, the distances showed an initial increase and then a subsequent decrease in these three directions, resulting in a funnel-shaped nodal distribution that more closely resembled an irregular diamond shape. The median and range of distances (in mm) from nodes to the nearest femoral artery, as well as margins for 90% and 95% coverage in different locations, are presented in Table 3.

Eighty-nine (18%) lymph nodes were located below the IPS, with a median distance of 17 mm (range 3–62) below the IPS, and the margin required to cover 95% of the lymph nodes was 26 mm below the IPS. Seventeen (3%) lymph nodes were located below the ILT, with a median distance of 10 mm (range 3–36) below the ILT, and the edge required to cover 95% of the lymph nodes was just at the level of ILT. The Median and range of distance (in mm) of nodes, as well as caudal edges of contouring for 90% and 95% nodal coverage from the IPS and the ILT, are displayed in Table 4.

**Table 3:** Median and range of distance (mm) of LNM to the nearest femoral artery and margins for 90% and 95% nodal coverage on a different location

Different locations	Lateral (10:00–11:00)			Anterior (12:00–1:00)			Medial (2:00–4:00)			
	Median (range) distance from femoral artery (mm)	Margin for 90% nodal coverage (mm)	Margin for 95% nodal coverage (mm)	Median (range) distance from femoral artery (mm)	Margin for 90% nodal coverage (mm)	Margin for 95% nodal coverage (mm)	Median (range) distance from femoral artery (mm)	Margin for 90% nodal coverage (mm)	Margin for 95% nodal coverage (mm)	
SFH	13 (6–20)	18	**19**	10 (3–20)	18	**19**	17 (6–30)	24	**25**	
SGT	16 (7–27)	23	**26**	11 (6–26)	19	**20**	16 (11–26)	21	**25**	
SPS	20 (10–30)	26	**28**	17 (6–37)	25	**29**	18 (7–31)	23	**26**	
IPS	20	-	**-**	20 (8–35)	27	**29**	19 (6–35)	25	**28**	
IIT	20	-	**-**	20 (10–43)	25	**27**	23 (10–30)	26	**27**	
ILT	20	-	**-**	19 (10–26)	23	**25**	20 (10–24)	22	**23**	

**Table 4:** Median and range of distance (mm) of LNM as well as caudal edges of contouring for 90% and 95% nodal coverage from the IPS and ILT

Reference axial planes	Median (range)distance from the axial plane	Inferior margin for 90% nodal coverage	Inferior margin for 95% nodal coverage
IPS	20 mm above this plane (the place below 62 mm from this plane to the place above 64 mm from this plane)	the place below 16 mm from this plane	the place below 26 mm from this plane
ILT	49 mm above this plane (the place below 36 mm from this plane to the place above 98 mm from this plane)	the place above 12 mm from this plane	on this plane

**Acronyms**: SFH = the level of the superior edge of the femoral head; SGT = the level of the superior edge of the greater trochanter; SPS = the level of the superior edge of the pubic symphysis; IPS = the level of the inferior edge of the pubic symphysis; IIT = the level of the inferior edge of the ischial tuberosity; ILT = the level of the inferior edge of the lesser trochanter

### Recommendations for CTV delineation of the inguinal lymphatic drainage area

Founded on the position information of LNM obtained from this study, the final recommended CTV for the inguinal lymphatic drainage area included cranial: the level of the SFH; caudal: the level of the ILT or 26 mm below the IPS; posterior: the posterior wall of the femoral vessel. For other directions, we suggest using different borders in different axial sections. The recommended distances from the femoral artery on the SFH were lateral 19 mm, anterior 19 mm, and medial 25 mm; on the SGT were lateral 26 mm, anterior 20 mm, and medial 25 mm; on the SPS were lateral 28 mm, anterior 29 mm, and medial 26 mm; on the IPS were lateral 0 mm, anterior 29 mm, and medial 28 mm; on the IIT were lateral 0 mm, anterior 27 mm, and medial 27 mm; on the ILT were lateral 0 mm, anterior 25 mm, and medial 23 mm. Use interpolation to contour the area between the 6 axial slices, and then the radiation oncologist should manually modify it in consideration of the specific situation of each level.

**Fig. 3:**
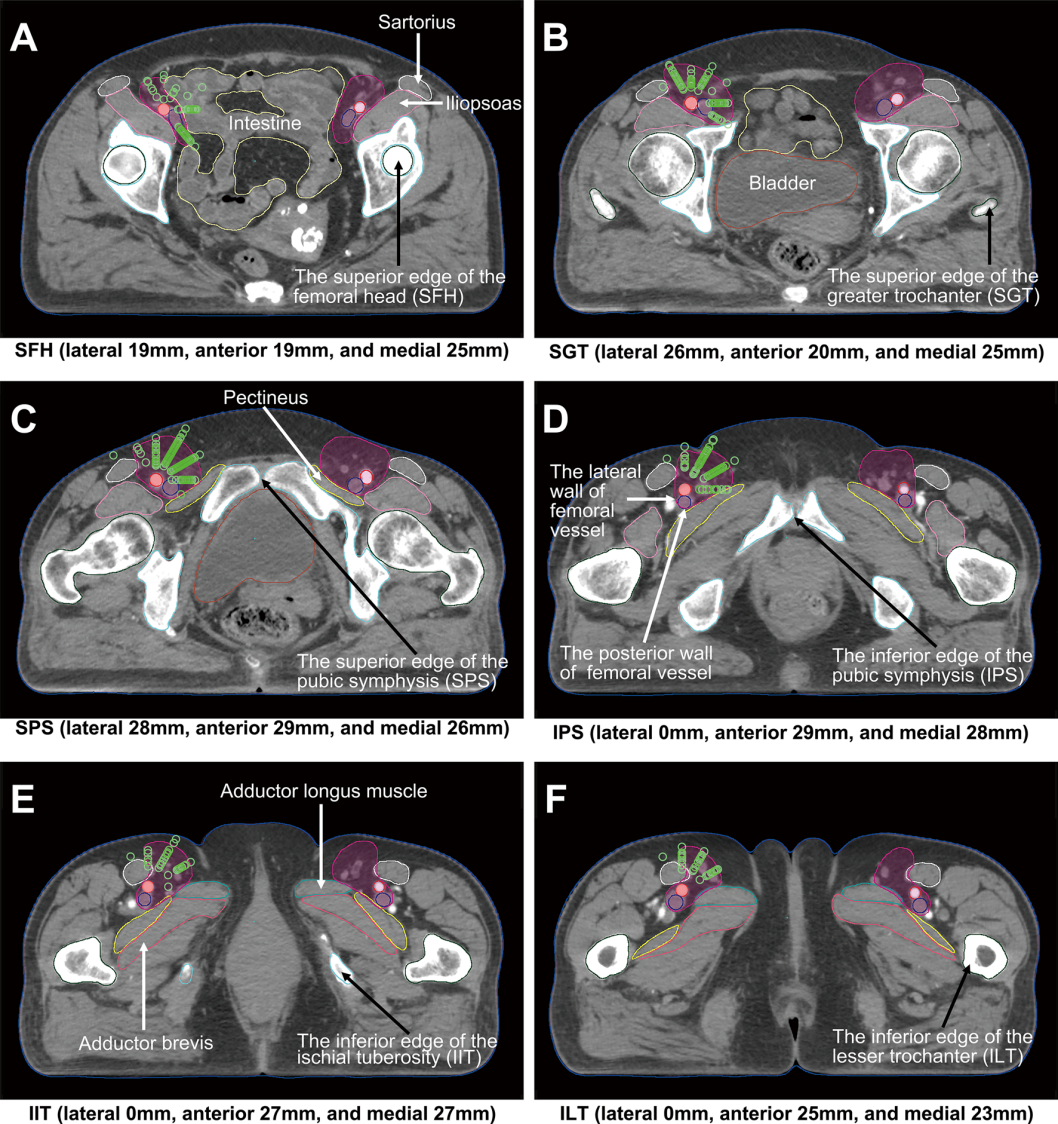
Distribution of lymph nodes and recommended inguinal CTV target delineation on different axial slices. The right inguinal area shows the distribution of lymph nodes (green circles) relative to the femoral artery (red). Our recommended inguinal CTV target delineation (purplish red filled area) concerning the femoral artery displays on both groin sides. The posterior border is defined by the posterior wall of the femoral vessels

All normal structures, such as the bowel, bladder, femoral head, femoral neck, and muscles, should be excluded from the CTV, while the femoral vessels-both femoral artery and vein should be included in the CTV. Furthermore, it is worth noting that only the levels involving and above the SPS are required to be delineated for the lateral region. And it should be mentioned that all clinically or radiographically suspicious lymph nodes need to be included, and the delineation should avoid extending beyond the body surface. Figure 3 illustrates the distribution of the lymph nodes and the recommended inguinal CTV target delineation on six representative axial CT planes.

## Discussion

The accuracy of CTV delineation is critical for achieving better outcomes in patients treated with IMRT. An accurate diagnosis of LNM is the first step in CTV delineation. Several studies have indicated that ^18^F-FDG PET/CT is one of the most accurate technologies for identifying subclinical LNM, with a sensitivity of more than 85% [16]. Among 185 patients with primary pelvic malignancies enrolled in this study, 500 metastatic inguinal lymph nodes were identified using ^18^F-FDG PET/CT. The use of PET/CT in radiotherapy planning resulted in the identification of more LNM compared to CT alone, thus improving the accuracy of CTV delineation. Our findings are consistent with those of DU et al., where changes in GTV delineation were observed in 10 (35.7%) patients derived from PET/CT information compared with CT data [25]. Similar studies on head, neck, thoracic, and pelvic tumors have demonstrated that PET/CT-guided radiotherapy is capable of optimizing the treatment volume, reducing radiotherapy toxicity, and improving overall patient survival [26–29]. Therefore, it is recommended that patients should undergo PET/CT examination before radiation treatment planning, if available. Hence, this study provides more reliable recommendations for inguinal CTV delineation using the advantages of PET/CT.

Prior to our study, four investigations assessed the distribution of inguinal lymph nodes in small cohorts of patients with various primary pelvic malignancies. Kim et al. [30] conducted a study with 22 patients, consisting of primary carcinomas originating in the pelvis, with 52 positive inguinal lymph nodes, while Rao et al. [31] studied 50 patients with primary carcinomas originating in the pelvis, with 150 positive inguinal lymph nodes. Both of these studies lacked precise localization of the lymph nodes, as they did not use clock-face directions or delineate the lymph node regions in a more detailed manner longitudinally. Garda et al. [32] were the first to utilize clock-face directions for accurate lymph node localization, but their study was limited to 40 patients with anal carcinoma, where a total of 79 radiographic LMN were identified by PET in 31 patients, CT in 28 patients, and MRI in 8 patients. Mittal et al. [33] investigated 23 patients with penile cancer and 222 inguinal lymph nodes. They introduced the use of the inferior pubic symphysis (IPS) to divide the lymph nodes longitudinally into upper and lower parts, recommending different target delineation ranges for each part. Chang et al. [34] studied 181 patients with pelvic malignancies and 415 involved inguinal lymph nodes treated with intensity-modulated radiation therapy. They divided the inguinal nodal CTV into three fields: horizontal superficial inguinal field (HSIF), vertical superficial inguinal field (VSIF); and deep inguinal field (DIF). The present study initially applied axial planes of six typical bony landmarks to locate lymph nodes, combining with a clockwise direction, enabling a more detailed CTV delineation. The femoral artery typically exhibits an approximate circle shape, serving as a convenient geographical reference for determining and identifying the distance and direction of the lymph node during data collection and CTV contouring.

In addition, with a clockwise direction of the right inguinal clock face representing the location of the lymph nodes, our findings in the present study indicated that LNM were distributed between 10:00 and 4:00, while no lymph nodes were distributed between 5:00 and 9:00. The overall distribution of LNM manifested a decreasing trend from anterior and medial regions of the level of SPS towards the periphery. In contrast to previous studies, our findings were narrower than Kim et al. [30], and wider than Rao et al. [31], as summarized in Supplementary Table 2. Additionally, Chang et al. [31] provided recommendations based on the distribution of lymph nodes relative to adjacent anatomical structures in the three regions. However, similar to the findings of these studies, our research also found that inguinal LNM were predominantly located medially or anteriorly to the femoral vessel, with rare occurrences in the lateral direction, and generally no lymph nodes were found behind the femoral vessel. In line with the results reported by Mittal et al. [33], the distribution of the inguinal lymph nodes was likened to a funnel.

After determining the distribution of the lymph nodes, we proceeded to delineate the inguinal target area. The data obtained suggested that as the femoral artery moved down, the distance from a node to the nearest femoral artery initially increased and then decreased. To minimize the target scope while ensuring adequate coverage, we recommended using different distances from the edge of the femoral artery in different directions for each axial slice. Specifically, on our designated six typical slices, the recommended distances were listed in the present study. Therefore, considering the distance from the lymph nodes to the femoral vessels, we conclude that the funnel-shaped nodal distribution more closely resembles an irregular diamond shape, which is a novel finding not mentioned in previous studies. Femoral vessels (including arteries and veins) are often considered high-risk areas, as they are accompanied by lymph pathways and communicate with lymph. Thus, they should be included in CTV delineation, irrespective of whether they are within the recommended distance or not.

Compared to our recommendations, the ranges recommended by guidelines or atlas for anal or rectal cancer [7–10, 12] were insufficient in the anterior and medial regions, while they were too wide in the posterior and lateral regions. Wright et al. [35] identified inguinal lymph node recurrence as the second most common site of local recurrence and proposed that the anterior border of the CTV should be extended further away from the femoral vessels. The anterior and medial edges recommended in our study are significantly larger than those in the guidelines [7–9, 12], and they overlap with the recommendations from similar studies mentioned in Supplementary Table 2.

Furthermore, considering that approximately half of the lymph nodes are distributed at the medial side of the femoral vessel, greater attention should be paid when delineating the medial area. Nilsson et al. [36] reported no instances of recurrence in the region posterolateral to deep femoral vessels after IMRT when CTV contained insufficient target volume in this area. We recommend using the lateral wall of the femoral vessel as the lateral border of the target volume, including and below the IPS, which is narrower than the medial edge of the sartorius or iliopsoas, as recommended by most guidelines [7, 9, 10]. For the posterior borders of the CTV, we suggest delimiting them by the posterior wall of the femoral vessel, excluding the region of the deep femoral vessel. This recommendation aligns with the consensus recommendations for vulvar carcinoma and the results of the most of studies, while being much smaller than the femoral triangle formed by iliopsoas, pectineus, and abductor longus muscles, as recommended by most contouring guidelines for rectal or anal cancer [7, 9–11].

As for the caudal edge of our recommended CTV, it was at the level of the ILT, or 26 mm below the IPS, providing coverage for 95% of the involved lymph nodes. Our recommended caudal edge was consistent with the UK recommendation [10], slightly lower than the level of IIT suggested by Australasian Gastrointestinal Trials Group [7], and 20 mm caudal to the saphenous-femoral junction recommended by Radiation Therapy Oncology Group [8]. Additionally, it was significantly lower than the level of saphenous-femoral junction recommended by International Consensus Guidelines in Rectal Cancer [9]. Concerning our findings, it appears that the current guidelines might pose a risk of inadequate treatment of subclinical metastatic lymph nodes and unnecessary radiation damage to surrounding tissue, which calls for caution among clinicians.

The median BMI of the patients in this study was 23.91, and the mean femoral vessel depth was 43 mm, which is similar to the patient whose template CT was used with a BMI of 24.44 and a femoral vessel depth of 46 mm. Therefore, the recommended margins in the groin we displayed are likely sufficient for subclinical metastatic lymph nodal coverage. However, it is essential to note that the anatomic relationships of vasculature and adjacent soft tissues in the groin vary by body weight and habitus, and some related studies have reported that femoral vessel depth is associated with the patient’s BMI [37]. As a result, radiologists should adapt the proposed delineation to suit the individual conditions of each patient.

As with other retrospective studies, certain limitations in our study should be acknowledged. First, the absence of pathological correlation for identified LNM could result in the misclassification of benign nodes as metastatic. Although ^18^F-FDG PET/CT, particularly with high SUV and distinct CT characteristics, has shown high diagnostic sensitivity and specificity in detecting metastatic lymph nodes, pathological confirmation would strengthen our findings. Second, our study assumes symmetrical lymph node enlargement centered on the original node for data consistency. However, this assumption may not hold true universally, potentially leading to bias in the measurement of node distribution and CTV delineation. Consequently, the actual microscopic extent of the disease may differ from our suggested boundaries. Finally, the generalizability of our findings is limited by anatomical variability and disease-specific differences across patient populations and tumor types. Further validation in more diverse populations is required to enhance the robustness of these conclusions.

## Conclusions

Inguinal LNM were predominantly located medially or anteriorly to the femoral artery, with only a few located laterally. Regarding the recommendation for inguinal nodal CTV delineation, we proposed using different borders in various axial planes and directions. In comparison with current guidelines and other studies, this study utilized ^18^F-FDG PET/CT to provide a more refined recommendation for inguinal CTV delineation.

## Electronic supplementary material

Below is the link to the electronic supplementary material.

Supplementary Material 1

Supplementary Material 2

## Data Availability

The datasets used and analyzed in this study are available from the corresponding author, Si-jin Li (Email: lisjnm123@163.com), upon reasonable request.

## Abbreviations

OS: Overall Survival
IMRT: Intensity Modulated Radiation Therapy
CTV: Clinical Target Volume
LNM: Lymph Node Metastases
^18^F-FDG: ^18^F-fluorodeoxyglucose
PET/CT: Positron Emission Tomography / Computed Tomography
BMI: Body Mass Index
SUVmax: Maximum Standardized Uptake Value
SUVmean: Mean Standardized Uptake Value
SFH: The Superior Edge of the Femoral Head
SGT: The Superior Edge of the Greater Trochanter
SPS: The Superior Edge of the Pubic Symphysis
IPS: The Inferior Edge of the Pubic Symphysis
IIT: The Inferior Edge of the Ischial Tuberosity
ILT: The Inferior Edge of the Lesser Trochanter
