# Supplementary material for: Investigation of the distribution of inguinal lymph nodes and delineation of the inguinal clinical target volume using 18F-FDG PET/CT
Source: BMC Cancer. 2024 Oct 10;24:1254. doi: 10.1186/s12885-024-13015-w (PMC11465914; doi:10.1186/s12885-024-13015-w)
Supplement: Supplementary file 1 — Supplementary Material 1 [file 12885_2024_13015_MOESM1_ESM.docx]

**Supplementary table1**

Published guidelines for delineation of inguinal lymph node CTV for radiotherapy

| Consensus Group | Cranial | Caudal | Anterior | Posterior | Lateral | Medial |
| --- | --- | --- | --- | --- | --- | --- |
| AGITG^#^ [7] | The level where the external iliac artery leaves the bony pelvis to become the femoral artery | The lower edge of the ischial tuberosities | A minimum 20-mm margin on the inguinal vessels, inclusive of any visible lymph nodes or lymphoceles | The bed of the femoral triangle is formed by the iliopsoas, pectineus, and adductor longus muscles | The medial edge of sartorius or iliopsoas | 10 to 20 mm margin around the femoral vessels (approximated by the medial third to half of the pectineus or adductor longus muscle) |
| RTOG^*^ [8] | The level of the caudad extent of the internal obturator vessels (approximate boney landmark: upper edge of the superior pubic rami) | 20 mm caudad to the saphenous/femoral junction | 7 to 8 mm margin in  soft tissue around the iliac vessels, consider larger (>10 mm) margin. Include any identified nodes | 7 to 8 mm margin in soft tissue around the iliac vessels, excluding bone and muscle. Include any identified nodes | 7 to 8 mm margin in soft tissue around the iliac vessels, consider larger (>10 mm) margin. Include any identified nodes | 7 to 8 mm margin in soft tissue around the iliac vessels, excluding bone and muscle. Include any identified nodes |
| International consensus guidelines in rectal cancer [9] | Where the deep circumflex vein crosses the external iliac artery. Alternatively (if  difficult detection on  CT images) between the acetabulum roof and the superior pubic rami | Where the great saphenous vein enters the femoral vein | At least 20 mm margin around inguinal vessels including any visible lymph nodes or lymphoceles | The femoral triangle formed by the iliopsoas, pectineus, and abductor longus muscles | The medial edge of the sartorius or iliopsoas muscles | 10-20 mm margin around the femoral vessels including any visible nodes or lymphoceles |
| UK National guidance for IMRT in anal cancer [10] | The external iliac nodal group | At the inferior slice demonstrating the lesser trochanter | Approximately 5 mm from the skin surface. All visible nodes and lymphoceles should be included | The pectineus, adductor longus, and ilio-psoas. All visible lymphoceles should be included | The medial edge of sartorius or ilio-psoas. All visible nodes and lymphoceles should be included | The spermatic cord in men, or the medial third to half of the pectineus or adductor longus muscle in women. All visible nodes and lymphoceles should be included |
| Consensus Recommendations of Vulvar Carcinoma ^[11]^ | Non-written description | Non-written description | Non-written description | The posterior of the femoral vessels | Non-written description | Non-written description |
| Target Volume Delineation  and Field Setup [12] | The external iliac nodal group | Non-written description | 10-15 mm around the vessels (excluding bone and muscle and skin) as well as any visualized lymph nodes in adjacent fat/soft tissues | 10-15 mm around the vessels (excluding bone and muscle and skin) as well as any visualized lymph nodes in adjacent fat/soft tissues | 10-15 mm around the vessels (excluding bone and muscle and skin) as well as any visualized lymph nodes in adjacent soft tissues | 10-15 mm around the vessels (excluding bone and muscle and skin) as well as any visualized lymph nodes in adjacent fat/soft tissues |

**Acronyms:** ^#^AGITG = Australasian Gastrointestinal Trials Group; ^*^RTOG = Radiation Therapy Oncology Group
