# Supplementary material for: Investigation of the distribution of inguinal lymph nodes and delineation of the inguinal clinical target volume using 18F-FDG PET/CT
Source: BMC Cancer. 2024 Oct 10;24:1254. doi: 10.1186/s12885-024-13015-w (PMC11465914; doi:10.1186/s12885-024-13015-w)
Supplement: Supplementary file 2 — Supplementary Material 2 [file 12885_2024_13015_MOESM2_ESM.docx]

**Supplementary table2**

Comparison of studies on contouring of inguinal clinical target volume

| **Study (patients)** | **Number of nodes** | **O’clock distribution**  **of nodes** | **The recommended margins (mm) around femoral vessels** | | | | | **Cranial edge** | **Caudal edge** |
| --- | --- | --- | --- | --- | --- | --- | --- | --- | --- |
|  |  |  | **Segment** | **Lateral** | **Anterior** | **Medial** | **Posterior** |  |  |
| Kim et al [27] (22 primary pelvic malignancies) | 52 | 9: 00-6:00 | All | ≥32 | ≥23 | ≥22 | ≥9 | Not reported | Not reported |
| Rao et al [28] (50 primary pelvic malignancies) | 150 | 11:00-3:00 | All | 27 | 22.5 | 15 | 0 | At the level where external iliac artery leaves the bony pelvis and becomes femoral artery | 20 mm below ILT |
| Garda et al [29] (40 anal squamous cell carcinoma) | 79 | 10:00-4:00 | All | 0 | 30 | 26 | 0 | At the level where external iliac artery leaves the bony pelvis and becomes femoral artery | 14 mm below IPS |
| Mittal et al [30] (33 penile squamous cell carcinoma) | 222 | 10:00-3:00 | supra-IPS | 21 | 29 | 23 | 0 | 48 mm above IPS | 50 mm below IPS |
|  |  |  | infra-IPS | 0 | 21 | 23 | 0 |  |  |
| Chang et al [31] (181 patients with pelvic malignancies) | 415 | - | HSIF | Medial edge of sartorius | Transverse fibrous septa within Camper’s fascia | 5 mm lateral to the anteromedial point of the superior pubic ramus | Scarpa’s fascia | Acetabulum roof | Saphenofemoral junction |
|  |  |  | VSIF | 20 mm lateral to the center of the great saphenous vein (GSV) | 18 mm anterior to the GSV | 10 mm medial to the center of the GSV | The posterior wall of the GSV | Saphenofemoral junction | Ischial tuberosity |
|  |  |  | DIF | 0 | Posterior border of the HSIF or VSIF | Pectineal muscle | Superior pubic ramus or pectineal muscle | Acetabulum roof | Saphenofemoral junction |
| This study (185 primary pelvic malignancies) | 500 | 10:00-4:00 | SFH | 19 | 19 | 25 | 0 | At the level of SFH | 26mm below IPS or at the level of ILT |
|  |  |  | SGT | 26 | 20 | 25 | 0 |  |  |
|  |  |  | SPS | 28 | 29 | 26 | 0 |  |  |
|  |  |  | IPS | 0 | 29 | 28 | 0 |  |  |
|  |  |  | IIT | 0 | 27 | 27 | 0 |  |  |
|  |  |  | ILT | 0 | 25 | 23 | 0 |  |  |

**Acronyms:** SFH=the level of the superior edge of the femoral head; SGT=the level of the superior edge of the greater trochanter; SPS=the level of the superior edge of the pubic symphysis; IPS=the level of the inferior edge of the pubic symphysis; IIT=the level of the inferior edge of the ischial tuberosity; ILT =the level of the inferior edge of the lesser trochanter
